# Biologic and targeted synthetic therapies in ankylosing spondylitis: a pharmacological review

**DOI:** 10.3389/fphar.2026.1865621

**Published:** 2026-08-26

**Authors:** Omar Z. Ameer, Reem Temsah, Yara O. Alsouss, Raghad Al-Amoudi, Mohammad A. Khanfar, Ibrahim M. Salman

**Affiliations:** 1 Department of Pharmaceutical Sciences, College of Pharmacy, Alfaisal University, Riyadh, Saudi Arabia; 2 Department of Biostatistics, Epidemiology and Public Health, College of Medicine, Alfaisal University, Riyadh, Saudi Arabia; 3 Department of Biology, College of Science, Northeastern University, Toronto, ON, Canada; 4 Department of Pharmacy Practice, College of Pharmacy, Alfaisal University, Riyadh, Saudi Arabia

**Keywords:** ankylosing spondylitis, biologics, IL-17 inhibitors, JAK inhibitors, targeted synthetic DMARDs, TNF inhibitors

## Abstract

Ankylosing spondylitis (AS) is a prototypical axial spondyloarthritis (axSpA) defined by chronic inflammatory back pain, progressive spinal stiffness, and frequent extra-articular manifestations such as acute anterior uveitis (21%–33%), inflammatory bowel disease (5%–10%), and psoriasis (4%–13%). Management has shifted from non-steroidal anti-inflammatory drugs (NSAIDs) to highly targeted biologic and synthetic disease-modifying antirheumatic drugs (DMARDs). This comprehensive review focuses on the current evidence for biologic and targeted synthetic therapeutics in the management of AS, emphasizing mechanisms of action, regulatory approvals, dosing regimens, and clinical efficacy. Three major therapeutic classes are identified: two biologic DMARD classes, comprising tumor necrosis factor-alpha (TNF-α) inhibitors and interleukin-17 (IL-17) inhibitors, and the targeted synthetic DMARDs, namely, the Janus kinase-signal transducer and activator of transcription (JAK-STAT) inhibitors. TNF inhibitors (adalimumab, infliximab, etanercept, golimumab, certolizumab pegol) typically achieve Assessment of Spondyloarthritis International Society 40% improvement (ASAS40) rates of 40%–58% at 12–24 weeks *versus* 13%–24% with placebo, and maintain clinical benefit in many patients over 5–8 years. IL-17 inhibitors (secukinumab, ixekizumab, bimekizumab, brodalumab, netakimab) yield broadly similar ASAS40 responses (30%–50% *versus* 10%–20% placebo) and are particularly useful in patients with concomitant psoriasis with substantially reduced risk of tuberculosis reactivation. JAK inhibitors (tofacitinib, upadacitinib) represent the newest class, offering oral administration with ASAS40 responses of 40%–52% *versus* 13%–26% for placebo in both biologic-naïve and biologic-experienced AS, though cardiovascular safety considerations necessitate careful patient selection. Therapeutic selection should consider comorbidities (uveitis favors TNF monoclonal antibodies; psoriasis favors IL-17 inhibitors), safety profile, route of administration, and sequencing after non-steroidal anti-inflammatory drug failure. Understanding these pharmacological distinctions enables personalized therapeutic decisions based on the specific molecular pathology and risk profile of the individual patient.

## 1 Introduction

Ankylosing spondylitis (AS) is the prototypical form of axial spondyloarthritis (axSpA), a chronic inflammatory disease of the axial skeleton that typically presents as inflammatory back pain before the age of 45 and may progress to structural damage and spinal fusion if left untreated (Papadakis et al., 2025). Within the axSpA spectrum, disease is classified as radiographic axSpA (r-axSpA), also termed AS, when characteristic sacroiliac joint or spinal changes are visible on plain radiographs, *versus* non-radiographic axSpA (nr-axSpA) when sacroiliitis and axial inflammation are detectable only by MRI (Papadakis et al., 2025; Robinson et al., 2021; Sieper and Poddubnyy, 2017).

Clinically, axSpA usually begins insidiously with morning stiffness, buttock or hip pain, and improvement with physical activity, features that help distinguish inflammatory from mechanical back pain (Ramiro et al., 2023). The inflammatory process often involves the sacroiliac joints and spine and may extend to peripheral sites, producing asymmetric oligoarthritis, enthesitis, and dactylitis that can precede overt radiographic spinal damage (Ramiro et al., 2023). Extra-musculoskeletal manifestations are common and clinically important: acute anterior uveitis occurs in roughly 21%–33% of patients (Rademacher et al., 2020), inflammatory bowel disease (IBD) in about 5%–10% (Llop et al., 2024), and psoriasis in approximately 4%–13% (Tian et al., 2023; Lucasson et al., 2022). Imaging is essential for confirming sacroiliac and spinal involvement and for early classification, given the overlap with mechanical back pain and the subtlety of early radiographic changes (Ramiro et al., 2023).

Generally, AS affects an estimated 0.2%–1.2% of the population, with a male-to-female ratio of roughly 2.5:1, reflecting a clear sex predominance (Protopopov and Poddubnyy, 2018; El, 2011). Genetic susceptibility is strongly linked to HLA-B27, which is present in up to 90% of White patients and around 50% of Black patients with axSpA (Sieper and Poddubnyy, 2017; Jamalyaria et al., 2017).The diagnostic value of HLA-B27 is constrained by its substantial prevalence in the general, unaffected population, which means a positive result is far from synonymous with the disease. In a large, nationally representative US survey, the age-adjusted prevalence of HLA-B27 among the general population was approximately 6.1%, reaching about 7.5% in non-Hispanic White individuals and roughly 3.5% across other race/ethnicity groups combined (Reveille et al., 2012). Marked geographical and ethnic variation is well recognized, with carriage frequencies ranging from very low levels in some populations to substantially higher levels in others, reflecting the marker’s heterogeneous global distribution and its numerous subtypes, only some of which are disease-associated (Khan, 2010). Given that the great majority of HLA-B27 positive individuals never develop axSpA, the marker has limited positive predictive value when applied indiscriminately; it is therefore most informative as part of structured probabilistic assessment, alongside inflammatory back pain, imaging, and other clinical features, rather than as a stand-alone diagnostic test (Reveille, 2015).

Prior to the introduction of biologic therapies, treatment options for AS were limited to non-steroidal anti-inflammatory drugs (NSAIDs, such as ibuprofen, naproxen, and the selective COX-2 inhibitor celecoxib), physiotherapy, and, in severe cases, conventional disease-modifying antirheumatic drugs (DMARDs such as methotrexate or sulfasalazine) with limited efficacy (Gravaldi et al., 2022; Ward et al., 2019; Danve and Deodhar, 2022).

Infliximab, the first approved tumor necrosis factor (TNF) inhibitor by both the European Medicines Agency (EMA) and the U.S Food and Drug Administration (FDA) for the treatment of active AS in 2003, marked a paradigm shift in disease management (Danve and Deodhar, 2022; Rudwaleit, 2010). Subsequently, multiple biologics targeting TNF-α and IL-17 pathways have demonstrated remarkable efficacy in controlling disease activity and improving functional outcomes (Alotaibi et al., 2024).

Although AS is part of a larger group of inflammatory disorders that fall under the “spondyloarthritis”, this review focuses on AS *per se* and its pathological features, treatment options with biologics, and their clinical characteristics.

This focused pharmacology review synthesizes current evidence on biologic and targeted synthetic therapies for AS, emphasizing mechanisms of action, regulatory approvals, dosing regimens, pivotal trial efficacy, long-term safety data, and practical treatment selection. Special attention is given to comorbidity-based drug positioning (uveitis, psoriasis, inflammatory bowel disease), sequencing strategies after NSAID failure, and comparative clinical positioning across therapeutic classes to support evidence-based, personalized therapeutic decision-making in AS management.

## 2 Methodology

This focused narrative review synthesizes evidence on biologic and targeted synthetic therapies for ankylosing spondylitis (AS). A structured literature search was conducted across PubMed (primary database), Google Scholar, and UpToDate, using controlled vocabulary and free-text terms including “ankylosing spondylitis,” “axial spondyloarthritis,” “TNF inhibitors,” “IL-17 inhibitors,” “JAK inhibitors,” “efficacy,” “safety,” “randomized trials,” “treatment sequencing,” and relevant drug names. Boolean operators (AND, OR) were applied, with reference lists hand-searched for additional studies. Inclusion prioritized pivotal phase II/III randomized controlled trials, long-term extension studies, real-world effectiveness data, systematic reviews/meta-analyses, and current treatment guidelines providing clinically actionable data on mechanisms, dosing, efficacy (ASAS40, BASDAI50, ASDAS), safety profiles, and comorbidity-based positioning. Studies were selected for contemporary relevance (post-2003 biologic era) while retaining foundational trials establishing class efficacy. Excluded were non-AS/axSpA studies (unless AS-specific data reported), non-biologic therapies (except treatment context), case reports/series, non-peer-reviewed material, and non-English publications lacking translation. The review emphasizes practical synthesis for evidence-based therapeutic decision-making, particularly treatment selection based on extra-articular manifestations (uveitis, psoriasis, IBD) and sequencing strategies.

**FIGURE 1:**
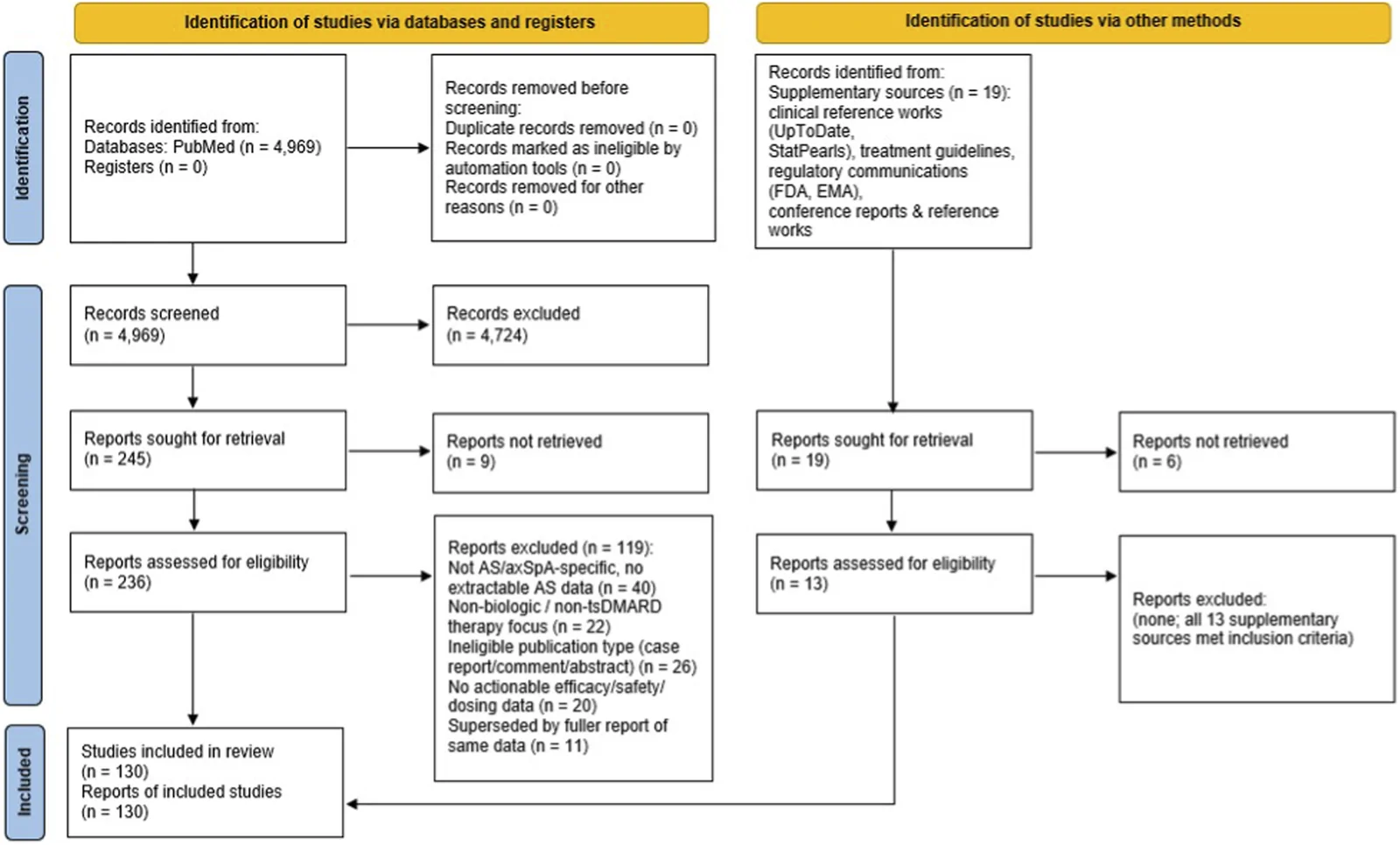
PRISMA 2020 flow diagram illustrating the selection of evidence on biologic and targeted synthetic DMARDs in ankylosing spondylitis. Records were identified through database searching (PubMed, Google Scholar, and UpToDate) and through additional sources including reference list screening and other relevant literature sources. Studies were screened and assessed for eligibility according to predefined inclusion and exclusion criteria before final inclusion in this review. The figure was created using the PRISMA 2020 Flow Diagram for New Systematic Reviews Which Included Searches of Databases, Registers and Other Sources template.

The study identification and selection process is summarized in Figure 1. A PRISMA 2020 flow diagram was used to document records identified through database searches and other sources, as well as the screening, eligibility assessment, and final inclusion of studies in this review. The figure was constructed using the PRISMA 2020 Flow Diagram for New Systematic Reviews Which Included Searches of Databases, Registers and Other Sources template.

To ensure contemporary relevance, the search emphasized recent literature and landmark trials, while still retaining older foundational studies that established key therapies or provided long-term safety and efficacy data. Priority was given to papers that directly informed current clinical practice, especially those describing approved biologics, emerging agents, or treatment selection based on comorbidities such as uveitis, psoriasis, and IBD. Overall, the review synthesizes evidence from the available literature to provide a clinically oriented overview of the evolving therapeutic options in AS.

## 3 Radiological features of ankylosing spondylitis

In AS, imaging is essential for diagnosis, disease monitoring, and treatment options. Conventional radiography and MRI provide a complementary insight into the disease. Although radiography lack sensitivity for early inflammation, it can detect late-stage structural damage such as sacroiliac erosions, sclerosis, ankylosis, and spinal syndesmophytes, which eventually give the appearance of a “bamboo spine.” On the other hand, MRI detects active inflammation, including bone marrow edema, which enables earlier diagnosis and treatment before irreversible structural damage occurs (Ostergaard and Lambert, 2012).

**FIGURE 2:**
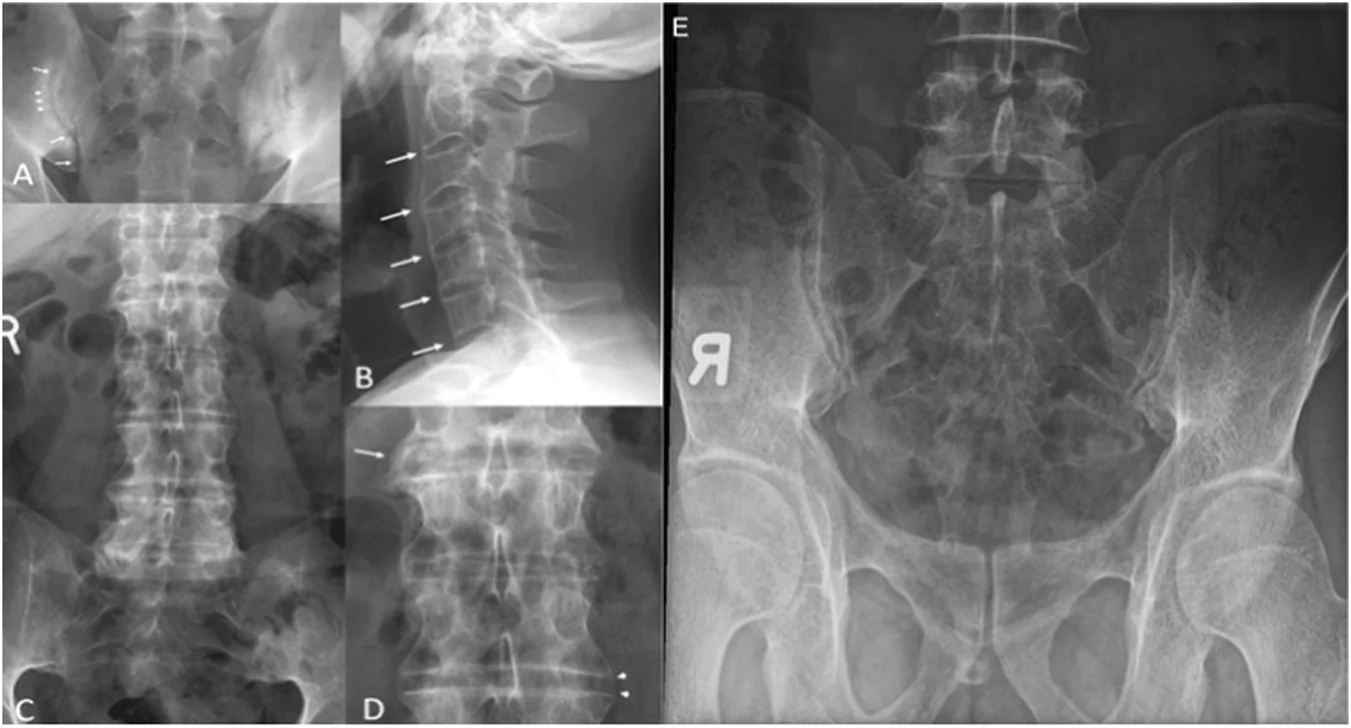
Representative radiographic features of ankylosing spondylitis. **(A)** Pelvic radiograph reveals bilateral sacroiliitis with erosions, poor joint definition, and adjacent subchondral sclerosis. **(B)** Cervical spine radiograph depicts vertically oriented syndesmophytes bridging anterior vertebral corners with facet joint fusion. **(C)** Lumbar spine radiograph indicates extensive vertebral ankylosis. **(D)** A detailed view of the lumbar spine highlighting syndesmophyte bridging and advanced ankylosis, resembling the “bamboo spine” appearance. **(E)** Normal sacroiliac joints with preserved joint space and smooth cortical margins (comparison image). Image is adapted and modified from (Ostergaard and Lambert, 2012). Note the letter ‘R’ (or ‘Я’) indicates the patient’s right side, as per radiographic orientation markers.

The disease’s characteristic radiological progression is shown in Figure 2. Panel A demonstrates bilateral sacroiliac joint pathology with erosions and subchondral sclerosis, indicating early involvement. As the disease progresses, syndesmophytes with bridging of the anterior vertebral borders occur, followed by vertebral body squaring and inflammation of the vertebral margins (Romanus lesions), as seen in panel B. Ankylosis is the final outcome of the progressive structural remodeling, demonstrated in panels C and D by extensive lumbar fusion with the characteristic “bamboo spine” appearance. For comparison, normal sacroiliac joints with preserved morphology are shown in panel E.

Axial spondyloarthritis (axSpA) is classified into radiographic (r-axSpA) and non-radiographic (nr-axSpA) forms. While r-axSpA shows sacroiliitis on X-ray, nr-axSpA is defined by the absence of radiographic changes and may require MRI for confirmation (Papadakis et al., 2025). Notably, 10%–40% of nr-axSpA cases progress to r-axSpA over 2–10 years (Protopopov and Poddubnyy, 2018). For clinicians, this distinction may help guide treatment decisions, as early MRI-detected inflammation predicts treatment response, whereas advanced radiographic damage reflects established disease (Ostergaard and Lambert, 2012).

Because plain radiographs are frequently unrevealing in early nr-axSpA, MRI of the sacroiliac joints has an important role in demonstrating active inflammatory change before definite structural abnormalities become apparent on radiography (Tsiami and Baraliakos, 2022). According to the ASAS and OMERACT consensus definition, a positive MRI requires definite bone marrow oedema or osteitis on a fluid sensitive sequence, such as STIR or T2 fat suppressed imaging, located in the subchondral or periarticular bone marrow and judged highly suggestive of axSpA; structural lesions alone, including erosion, fat metaplasia, sclerosis, or ankylosis, and isolated synovitis, enthesitis, or capsulitis are not sufficient in the absence of bone marrow oedema (Lambert et al., 2016). Since low grade sacroiliac marrow oedema may also occur in individuals without axSpA, more specific data driven MRI lesion thresholds have been proposed for early disease. Analyses from the ASAS MRI working group showed that bone marrow oedema is highly suggestive of axSpA when it involves at least four sacroiliac joint quadrants or is present in the same location on at least three consecutive slices (Krabbe et al., 2020). Structural lesions considered highly suggestive of axSpA included erosion involving at least three sacroiliac joint quadrants or the same location on at least two consecutive slices, and fat lesion involving at least five sacroiliac joint quadrants or the same location on at least three consecutive slices (Maksymowych et al., 2021). Data-driven MRI analyses in juvenile spondyloarthritis have identified quantitative thresholds for sacroiliac joint lesions typical of axial disease. Inflammatory involvement is defined by bone marrow edema affecting at least three sacroiliac joint quadrants, with a sensitivity of 98.6% and specificity of 96.5%. Structural involvement is defined by erosion in at least three quadrants or by sclerosis, fat lesions, backfill, or ankylosis affecting at least two quadrants or joint halves, with a sensitivity of 98.6% and specificity of 95.5% (Weiss et al., 2023). These thresholds may therefore be used as supportive baseline MRI markers in suspected early nr-axSpA, but they should always be interpreted in conjunction with the overall clinical context rather than in isolation (Tsiami and Baraliakos, 2022).

### 3.1 The modified New York criteria and the classification of radiographic axial spondyloarthritis

Although MRI now permits earlier detection of axial inflammation, the modified New York criteria remain historically foundational and clinically relevant to the classification of r-axSpA. Proposed in 1984 as a refinement of the original 1966 New York criteria, they require the presence of definite radiographic sacroiliitis together with at least one clinical criterion in order to classify a patient as having definite AS (van der Linden et al., 1984). The radiographic component is graded on a 0–4 scale, and the threshold for definite sacroiliitis is bilateral grade ≥2 changes or unilateral grade 3–4 changes on plain pelvic radiographs. The accompanying clinical criteria are inflammatory low back pain persisting for at least 3 months that improves with exercise but not with rest, limitation of lumbar spine motion in both the sagittal and frontal planes, and reduced chest expansion relative to age and sex matched reference values (van der Linden et al., 1984). Because these criteria depend on structural damage that becomes visible only after a prolonged period of inflammation, they are insensitive to early disease and do not capture patients with nr-axSpA, in whom sacroiliitis may be detectable by MRI but not yet by radiography. This limitation was a major driver for the development of the broader ASAS classification criteria, which incorporate MRI and HLA-B27 to identify the full axSpA spectrum at an earlier stage (Rudwaleit et al., 2009). Nevertheless, the modified New York criteria remain important because they continue to define the radiographic end of the axSpA spectrum and are still widely used as a reference standard in epidemiological studies and clinical trials (Ramiro et al., 2023; van der Linden et al., 1984).

## 4 Pathogenesis of ankylosing spondylitis

**FIGURE 3:**
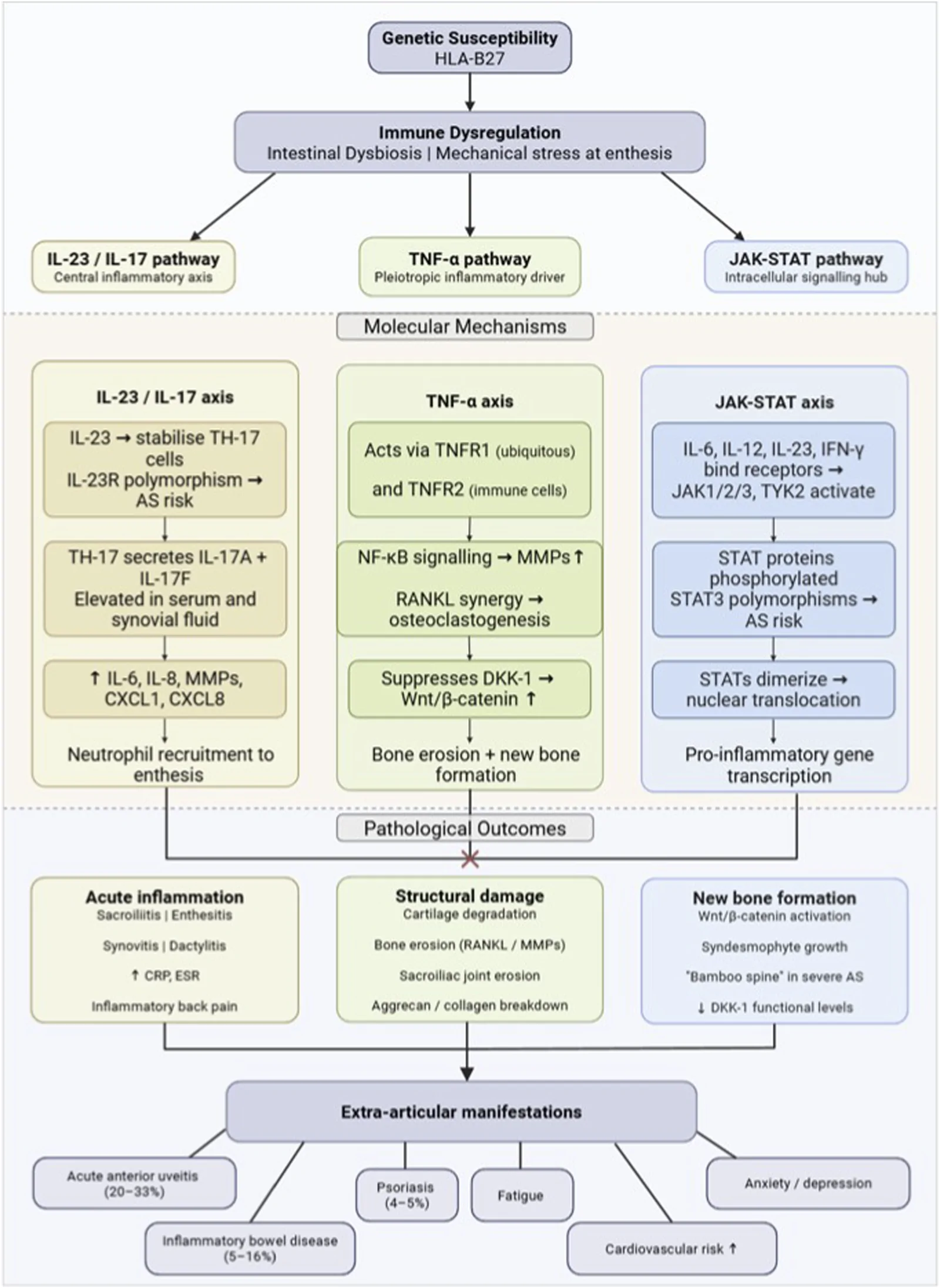
Major molecular signaling involved in the pathogenesis of ankylosing spondylitis. Schematic overview of the key molecular pathways driving inflammation and structural damage in ankylosing spondylitis. Genetic susceptibility, primarily conferred by HLA-B27, triggers immune dysregulation through intestinal dysbiosis and mechanical stress at entheses, activating three major inflammatory axes: the IL-23/IL-17 pathway, the TNF-α pathway, and the JAK-STAT signaling pathway. Downstream pathological outcomes include acute inflammation, structural bone damage, and pathological new bone formation, collectively contributing to extra-articular manifestations. Adapted and modified (Simone et al., 2018; Toussirot, 2022; Jethwa and Bowness, 2016; Lata et al., 2019). Figure created using software tools of BioRender.com.

The pathogenesis of AS involves a complex interplay between genetic predisposition, particularly the strong association with HLA-B27, and immune dysregulation (Figure 3). Three therapeutic target pathways predominate including the IL-23/IL-17 axis, tumor necrosis factor-alpha (TNF-α) signaling, and JAK-STAT cascades (Simone et al., 2018; Toussirot, 2022).

IL-23/IL-17 Axis: IL-23 stabilizes Th17 cells and innate lymphoid cells. This drives IL-17A/F secretion. These cytokines stimulate mesenchymal cells including osteoblasts and fibroblasts to release IL-6, IL-8, and matrix metalloproteinases (MMPs). Consequently, neutrophils recruit and promote enthesitis, synovitis, and osteoproliferation (Simone et al., 2018; Jethwa and Bowness, 2016).

Nonetheless, therapeutic targeting of this pathway in AS has revealed an important biological paradox. Despite strong genetic associations with IL23R and compelling preclinical evidence implicating the IL-23/IL-17 axis in disease pathogenesis, selective inhibition of IL-23 has failed to demonstrate meaningful clinical efficacy in axSpA. Clinical trials evaluating the IL-23 p19 inhibitors risankizumab and tildrakizumab, as well as IL-12/23 p40 blockade, did not improve disease activity, despite the well-established efficacy of these agents in psoriasis and psoriatic arthritis. Current evidence suggests that although IL-23 may contribute to disease initiation, established axial disease is maintained by multiple innate and adaptive immune cell populations, including entheseal resident γδ T cells, mucosal-associated invariant T (MAIT) cells, and innate lymphoid cells, which can produce IL-17 independently of IL-23 signaling. This apparent IL-23 paradox indicates that the IL-23/IL-17 axis is more complex than a simple linear pathway. It also provides a biological explanation for why direct IL-17 inhibition has consistently demonstrated clinical efficacy in AS, whereas selective IL-23 blockade has not. These findings highlight IL-17 as the principal effector cytokine driving established axial disease and emphasize the importance of distinguishing mechanisms involved in disease initiation from those responsible for disease maintenance (McGonagle et al., 2021; Zhang et al., 2022).

TNF-α Pathway: This pleiotropic cytokine mediates acute inflammation and osteoclastogenesis (bone erosion) *via* RANKL/TNFR1 signaling. Paradoxically, TNF-α suppresses Wnt inhibitor Dickkopf-1 (DKK-1). This enhances osteoblast activity and syndesmophyte formation explaining initial erosion followed by ankylosis (Lata et al., 2019).

JAK-STAT Signaling: This serves as intracellular hub for IL-6, IL-23, interferon gamma, and other cytokines. It amplifies Th17 differentiation and inflammatory gene transcription thus rationalizing small molecule inhibitor efficacy (Toussirot, 2022), (Figure 3).

## 5 Pharmacology of biologics in ankylosing spondylitis

### 5.1 TNF-α inhibitors

#### 5.1.1 Mechanism of action

The cytokine TNF-α plays a multifaceted role in AS pathogenesis, contributing to inflammation, bone erosion, and paradoxically to new bone formation (Lata et al., 2019). Early-stage AS is dominated by inflammatory processes characterized by back pain, joint swelling, and elevated acute-phase reactants, with TNF-α abundant in sacroiliac joints (Lin et al., 2021). Evidently, TNF-α mediates its effects through two receptors: TNFR1 (widely expressed) and TNFR2 (primarily on immune cells) (Lata et al., 2019).​ During the inflammatory phase, TNF-α synergizes with receptor activator of nuclear factor-κB ligand (RANKL) to enhance osteoclastogenesis, promoting local bone erosion (Luo et al., 2018). Moreover, TNF-α induces expression of MMPs and aggrecanases which degrade collagen, aggrecan, and other cartilage components (Xue et al., 2013). These catabolic effects are primarily mediated through TNFR1 *via* nuclear factor-κB (NFκB) signaling (Lata et al., 2019).

**FIGURE 4:**
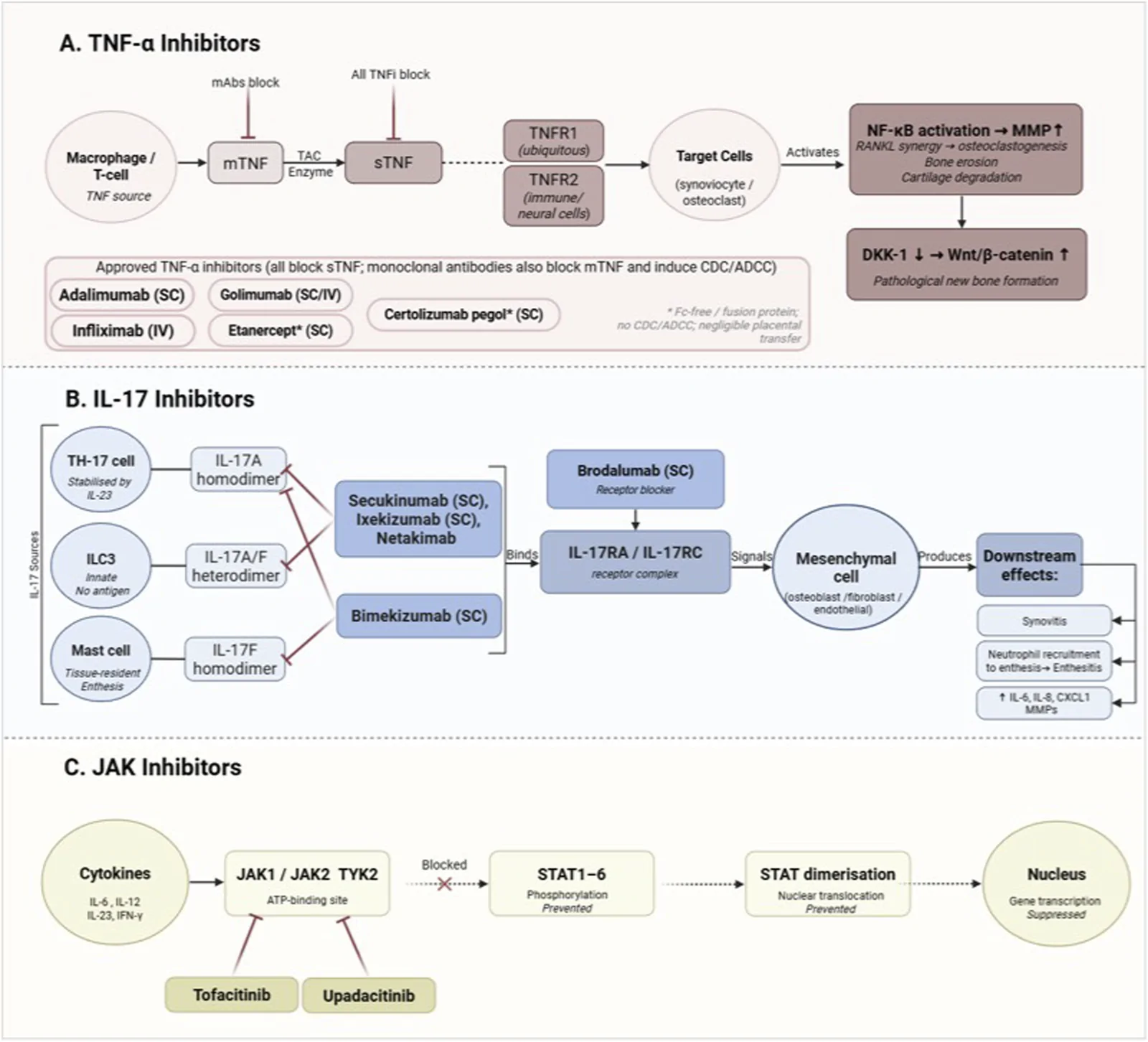
Mechanism of action of DMARDs: targeted biologic and targeted synthetic DMARDs in Ankylosing Spondylitis. **(A)** Illustrates TNF-α inhibitors, which block membrane-bound TNF (mTNF) and/or soluble TNF (sTNF), generated by TACE-mediated cleavage from macrophages and T-cells, therefore preventing downstream NF-κB-driven inflammation, RANKL-mediated osteoclastogenesis, and DKK-1 suppression-induced pathological new bone formation *via* Wnt/β-catenin signalling (Lata et al., 2019). **(B)** Illustrates IL-17 inhibitors, which intercept cytokines secreted by Th17 cells, ILC3s, and mast cells at entheseal sites; ligand blockers (secukinumab, ixekizumab, netakimab) neutralise IL-17A, bimekizumab provides dual IL-17A/F blockade, and brodalumab blocks the IL-17RA receptor, collectively preventing downstream pro-inflammatory mediator release, neutrophil recruitment, and entheseal damage (McGonagle et al., 2019; Tam et al., 2022; Pavelka et al., 2020; Blair, 2019). **(C)** Illustrates JAK inhibitors (tofacitinib, upadacitinib), which competitively block the ATP-binding site of receptor-associated JAK1/JAK2/TYK2, preventing phosphorylation and nuclear translocation of STAT proteins and suppressing pro-inflammatory gene transcription; all downstream steps shown in grey represent prevented consequences of JAK blockade rather than independent drug targets (Toussirot, 2022; Jethwa and Bowness, 2016). Figure is adapted and modified from the stated references and created using software tools of BioRender.com. ADCC, antibody-dependent cellular cytotoxicity; CDC, complement-dependent cytotoxicity; DMARDs, Disease-Modifying Antirheumatic Drugs; DKK-1, Dickkopf-1; ILC3, innate lymphoid cell type 3; JAK, Janus kinase; MMP, matrix metalloproteinase; NF-κB, nuclear factor kappa B; RANKL, receptor activator of NF-κB ligand; STAT, signal transducer and activator of transcription; TACE, TNF-α converting enzyme; TNF, tumor necrosis factor; TNFR, TNF receptor; and TYK2, tyrosine kinase 2.

Paradoxically, TNF-α also contributes to pathological new bone formation in AS. Although TNF-α is classically associated with inflammation and bone resorption, it also participates in the dysregulation of osteogenic signaling pathways characteristic of AS. In this context, reduced functional activity of DKK-1, a key antagonist of the Wnt pathway, results in inadequate suppression of Wnt/β-catenin signaling, thereby favoring osteoblast differentiation and activity. Lower functional DKK-1 levels correlate with syndesmophyte development in AS patients. Additionally, TNF-α increases expression of WNT5a and promotes tissue non-specific alkaline phosphatase-mediated mineralization in mesenchymal stem cells. This dual role explains the characteristic pattern of AS where initial inflammation and erosion are followed by excessive new bone formation, unlike the predominantly erosive pattern in rheumatoid arthritis (Lata et al., 2019), (Figure 4A).

#### 5.1.2 Clinical efficacy

A 2015 Cochrane systematic review and meta-analysis of 21 trials including 3,308 participants showed that anti-TNF agents improved clinical outcomes in ankylosing spondylitis, with patients receiving active treatment being about three to four times more likely to achieve an ASAS40 response by 6 months than those receiving placebo. In that analysis, adalimumab, etanercept, golimumab, and infliximab all demonstrated significant efficacy, with indirect comparisons suggesting no clear class superiority in short term response (Maxwell et al., 2015). Table 1 provides an overview of these agents, highlighting their molecular structures, pharmacokinetic profiles, and dosing regimens, alongside key clinical outcomes and approval status for the treatment of AS. Adalimumab, etanercept, and infliximab have also shown clinically meaningful improvements in observational and trial settings, with ASAS40 response rates commonly in the 40%–60% range during the first 12–24 weeks of treatment (Sieper et al., 2012; Peyrin-Biroulet et al., 2021; Yuk et al., 2024). Long term extension studies confirm sustained benefit with TNF inhibition. In the 5-year ATLAS extension, adalimumab maintained durable efficacy, and among patients who completed 5 years of continuous therapy, 70% achieved ASAS40, 77% achieved BASDAI50, 51% achieved ASAS partial remission, and 61% achieved ASDAS inactive disease. Early clinical response at 12 weeks was the strongest predictor of sustained long-term remission (Sieper et al., 2012). Similarly, infliximab demonstrated prolonged effectiveness in the European AS Infliximab Cohort, with stable disease control over 8 years and the feasibility of extending infusion intervals in selected patients with maintained clinical benefit (Baraliakos et al., 2016).

**TABLE 1:** TNF-α inhibitors in ankylosing spondylitis.

Drug	Structure	MW (kDa)	MOA	Dose	PK	Key outcomes	Notes	FDA approval	References
Adalimumab	Fully human IgG1 mAb	148	Binds soluble and membrane bound TNF-α	40 mg SC Q2W	t_1/2_ 10–20 days; peak: 131 ± 56 h; BA 64%; steady-state 12–20 weeks	ASAS40: 70%, BASDAI50: 51%, ASAS partial remission: 51%, ASDAS inactive disease: 61% ASAS20: 58.2% vs. 20.6%; BASDAI50: = 45.2% vs. 15.9% at week 12 in patients with active AS (ATLAS; n = 315)	CDC and ADCC Strong efficacy in uveitis and IBD; sustained remission up to 5 years	2006	(Sieper et al., 2012; van der Heijde et al., 2006; van der Heijde et al., 2009)
Infliximab	Chimeric mAb, 75% human and 25% murine	149	Binds soluble and membrane bound TNF-α	5 mg/kg IV at 0, 2, and 6 weeks, then Q6W-Q8W	t_1/2_ 8–12 days; steady-state 14 weeks	ASAS20: 61.2% vs. 19.2% at week 24 in active AS (ASSERT; n = 279)	CDC and ADCC; strong efficacy in uveitis and IBD; high immunogenicity with anti-drug antibodies reported in 28% of IBD patients	2004	(Baraliakos et al., 2016; van der Heijde et al., 2005)
Golimumab	Fully human IgG1 mAb	150	Binds soluble and membrane bound TNF-α	50 mg SC monthly or 2 mg/kg IV	t_1/2_ about 2 weeks	ASAS20: 59%–60% vs. 21.8% at week 14 in active AS (GO-RAISE; n = 356) ASAS40 57% at 5 years	CDC and ADCC; effective in uveitis and IBD	2009	(Braun et al., 2012; Deodhar et al., 2015)
Etanercept	TNFR2-IgG1 Fc fusion protein	150	Acts as TNF receptor decoy	50 mg SC weekly or 25 mg twice weekly	t_1/2_ about 70 h; peak concentration after 48 h; BA 76%	ASAS20: 59% vs. 28% at week 12 and 57% vs. 22% at week 24 in active AS	Faster binding kinetics; reduced efficacy in uveitis	2003	(Davis et al., 2003)
Certolizumab pegol	Pegylated Fab fragment lacking the Fc portion	91	Fab only	400 mg at 0, 2, and 4 weeks, then 200 mg Q2W or 400 mg Q4W	t_1/2_ 14 days	ASAS20: 57.7%–63.6% vs. 38.3% at week 12 in axSpA (RAPID-axSpA; n = 325)	Low placental transfer; PEG prolongs t1/2	2013	(Sieper et al., 2015)

This table summarizes the pharmacologic profiles and clinical efficacy of TNF-α inhibitors, which were the first biologics to revolutionize AS management. These agents generally produce ASAS20 responses within 12–24 weeks and can maintain clinical benefit for several years, with class selection often guided by route of administration, immunogenicity, and extra articular manifestations.

ADCC, antibody-dependent cellular cytotoxicity; AS, ankylosing spondylitis; ASAS20, Assessment of Spondyloarthritis International Society 20%; ASAS40, Assessment of Spondyloarthritis International Society 40%; ASAS, Assessment of Spondyloarthritis International Society; ASDAS, Ankylosing Spondylitis Disease Activity Score; ASSERT, Ankylosing Spondylitis Study for the Evaluation of Recombinant Infliximab Therapy; ATLAS, Adalimumab Trial Long-term Ankylosing Spondylitis; axSpA, axial spondyloarthritis; BA, bioavailability; BASDAI50, Bath Ankylosing Spondylitis Disease Activity Index 50%; CDC, complement-dependent cytotoxicity; Fab, fragment antigen-binding; Fc, fragment crystallizable; FDA, Food and Drug Administration; hr, hour; IBD, inflammatory bowel disease; IgG1, immunoglobulin G1; IV, intravenous; kDa, kilodalton; mAb, monoclonal antibody; mg, milligram; mg/kg, milligram per kilogram; MOA, mechanism of action; MW, molecular weight; n, sample size; PEG, polyethylene glycol; PK, pharmacokinetics; Q2W, every 2 weeks; Q4W, every 4 weeks; Q6W, every 6 weeks; Q8W, every 8 weeks; RR, relative risk; SC, subcutaneous; t1/2, half-life; TNF-α, tumor necrosis factor-alpha; TNFR2, tumor necrosis factor receptor 2.

#### 5.1.3 Safety considerations

TNF monoclonal antibodies, including adalimumab, infliximab, and golimumab, appear more effective than etanercept for preventing recurrent uveitis, likely because monoclonal antibodies can target membrane bound TNF more effectively and mediate complement dependent cytotoxicity (Zhao X. et al., 2025; Ahn et al., 2022; Arora et al., 2009). Real world studies also suggest lower rates of uveitis with monoclonal antibodies than with etanercept (Ahn et al., 2022). TNF monoclonal antibodies and certolizumab pegol are effective in both AS and inflammatory bowel disease, particularly Crohn’s disease, whereas etanercept is effective for AS but is not useful for inflammatory bowel disease (Peyrin-Biroulet et al., 2021; Marin et al., 2018; Schreiber, 2011).

Because TNF alpha is important for granuloma maintenance, TNF inhibitors increase the risk of latent tuberculosis reactivation, especially in endemic regions (Yuk et al., 2024; Alıravcı et al., 2025). Baseline screening with a tuberculin skin test or interferon gamma release assay, chest radiography, and exposure history are recommended before treatment, with periodic reassessment in high-risk settings (Shim, 2014; Maeda et al., 2024). Serious infection rates in AS trials have generally been low, and other adverse events such as injection site reactions are usually mild to moderate (Mounach and El Maghraoui, 2014; Feng et al., 2023). Long term safety data for adalimumab shows low malignancy rates, excluding lymphoma and non-melanoma skin cancer (Mounach and El Maghraoui, 2014). Biosimilar formulations of adalimumab, infliximab, and etanercept have shown therapeutic equivalence and broaden access to treatment (Ascef et al., 2023).

### 5.2 Interleukin-17 inhibitors

#### 5.2.1 Mechanism of action

Interleukin-17 inhibitors represent an important advance in the treatment of AS by targeting the IL-23/IL-17 axis at the level of the cytokine ligand or receptor. The biologic rationale is supported by evidence showing that IL-17A and IL-17F are elevated in the serum, synovial fluid, and affected tissues of patients with AS (McGonagle et al., 2019). These cytokines promote inflammation by inducing IL-6, IL-8, chemokines, and MMPs, and by enhancing neutrophil recruitment at sites of entheseal inflammation. Genetic studies have also identified IL-23R polymorphisms associated with disease susceptibility, further supporting the relevance of this pathway in AS pathogenesis (Layh-Schmitt and Colbert, 2008), (Figure 4B).

Ligand binding agents such as secukinumab and ixekizumab are monoclonal antibodies that selectively neutralize IL-17A homodimers and IL-17A/F heterodimers, thereby preventing interaction with the IL-17 receptor complex (IL-17RA/RC) (Tam et al., 2022). This blocks downstream chemokine release, including CXCL1 and CXCL8, and reduces neutrophil driven inflammation at the entheses (Matsuzaki et al., 2015), (Figure 4B). Bimekizumab has a dual variable domain design that enables simultaneous neutralization of IL-17A and IL-17F isoforms (van der Heijde et al., 2020). This dual blockade is pharmacologically relevant because IL-17F shares structural similarity with IL-17A and can maintain inflammatory signaling when IL-17A alone is inhibited (Glatt et al., 2018). In contrast, brodalumab binds to IL-17RA and blocks receptor mediated signaling. Because IL-17RA is shared by several IL-17 family cytokines, including IL-17A, IL-17F, IL-17C, IL-17E, and IL-17A/F heterodimers, brodalumab provides the broadest inhibition of IL-17 family signaling (Wei et al., 2021).

#### 5.2.2 Clinical efficacy

The MEASURE trials established secukinumab as the first IL-17A inhibitor approved for AS (Braun et al., 2017; Marzo-Ortega et al., 2020a; Pavelka et al., 2017; Kivitz et al., 2018). In MEASURE 1 and MEASURE 2, which included 590 patients, ASAS20 response at week 16 was approximately 60%–61% with secukinumab 150 mg and 75 mg compared with 29% with placebo, while ASAS40 response was approximately 28%–36% *versus* 11% with placebo (Braun et al., 2017; Marzo-Ortega et al., 2020a; Baeten et al., 2015). Two-year data from MEASURE 1 confirmed sustained benefit, with a mean change in modified Stoke AS Spinal Score (mSASSS) from baseline to week 104 of 0.30 ± 2.53, suggesting low average radiographic progression. Among patients with evaluable radiographs, ASAS20 response in week 104 was 73.7% in the 150 mg group and 68.0% in the 75 mg group (Braun et al., 2017). In MEASURE 3, secukinumab with an intravenous loading regimen followed by subcutaneous maintenance also showed durable efficacy, with ASAS20 response at week 16 of 58.1% with 150 mg and 60.5% with 300 mg *versus* 36.8% with placebo, and sustained responses through week 156 in long term follow up (Pavelka et al., 2020; Pavelka et al., 2017). Table 2 provides additional detail on major clinical outcomes, dosing regimens, regulatory approvals, and other core features specific to each agent in this class.

**TABLE 2:** IL-17 inhibitors in ankylosing spondylitis.

Drug	Structure	MW (kDa)	MOA	Dose	PK	Key outcomes	Notes	FDA approval	References
Secukinumab	Fully human IgG1κ mAb	148	Selectively neutralizes IL-17A	150 mg SC; initial dosing starting at weeks 0–4, then once monthly; may increase to 300 mg SC Q4W if disease activity remains high; IV loading 6 mg/kg at week 0 followed by 1.75 mg/kg Q4W, maximum 300 mg per infusion	t_1/2_ 27 days; Vd 7.1–8.6 L; steady-state: week 24	ASAS20 60%–61% and ASAS40 28%–36% at week 16 in active AS in the MEASURE trials; sustained efficacy to 5 years, with MEASURE 1 showing ASAS20 78.6% and ASAS40 65.2%; and at week 104 ASAS20: 73.7% (150 mg) and 68.0% (75 mg) MEASURE 3 achieved ASAS20 at week 16: 58.1% (150 mg) and 60.5% (300 mg)	Low immunogenicity	2016	(Pavelka et al., 2020; Braun et al., 2017; Pavelka et al., 2017; Baraliakos et al., 2019)
Ixekizumab	Humanized IgG4 mAb	146.16	Selectively neutralizes IL-17A	160 mg SC initial dose as two 80 mg injections at week 0, then 80 mg Q4W	t_1/2_ 13 days; BA 60%–81%; peak concentration about 4 days after dose; Vd 7.11 L	ASAS40 48%–52% vs. 19% placebo at week 16 in biologic-naïve active r-axSpA in COAST V (n = 341) ASAS40 response in COAST-W at week 16: 25%–31%	Low CDC and ADCC	2019	(Dougados et al., 2020; van der Heijde et al., 2018a)
Netakimab	Humanized mAb	145.74	IL-17A blockade	120 mg SC at weeks 0, 1, and 2, then Q2W	Not reported in published literature	ASAS40 rates of 40.91%–72.73% vs. 14.29% placebo at week 16 in phase 2 active AS despite NSAIDs; and in the phase 3 ASTERA trial ASAS40 reached 80.7% at week 52	Fastest response at 120 mg; dose dependent efficacy and favorable safety profile	Not currently FDA-approved, as it has not been submitted for FDA evaluation. It was developed and marketed in Russia; therefore, it is only approved for the treatment of AS in Russia and Belarus	(Erdes et al., 2020; Erdes et al., 2021; Mazurov et al., 2020)
Bimekizumab	Humanized IgG1 mAb	147.2	Dual neutralization of IL-17A and IL-17F	160 mg SC Q4W	t_1/2_ 23 days; Vd 11.2 L	ASAS40 response at week 16 in BE MOBILE 1 and 2: 47.7% in nr-axSpA and 44.8% in r-axSpA ASAS40 29.5%–46.7% vs. 13.3% placebo in BE AGILE; sustained ASAS40 58.6%–62.3% at week 48	Deeper suppression of inflammation due to dual targeting	2024	(van der Heijde et al., 2023; Baraliakos et al., 2024a)
Brodalumab	Fully human IgG2κ mAb	144	Binds IL 17RA and blocks signaling by multiple IL 17 family cytokines	210 mg SC Q2W	t_1/2_ 10.9 days; Vd 8.9 L	ASAS40 43.8% vs. 24.1% at week 16 in active axSpA; sustained improvements through 68 weeks	Broadest blockade of IL-17 signaling	Not approved by the FDA for AS since it did not undergo an evaluation for this specific indication. Moreover, previous safety concerns regarding suicidal ideation may have influenced this decision	(Wei et al., 2021; Roman and Chiu, 2017)

This table summarizes the IL-17 inhibitor class, including ligand blockers, dual IL-17A/F blockade, and receptor blockade. These agents produce ASAS40 responses of roughly 30%–50% in pivotal trials, with a generally favorable safety profile and lower risk of tuberculosis reactivation than TNF inhibitors.

ADCC, antibody-dependent cellular cytotoxicity; AS, ankylosing spondylitis; ASAS20, Assessment of Spondyloarthritis International Society 20%; ASAS40, Assessment of Spondyloarthritis International Society 40%; axSpA, axial spondyloarthritis; BA, bioavailability; CDC, complement-dependent cytotoxicity; FDA, Food and Drug Administration; IgG1, immunoglobulin G1; IgG2, immunoglobulin G2; IgG4, immunoglobulin G4; IL-17A, interleukin-17A; IL-17RA, interleukin-17 receptor A; IV, intravenous; kDa, kilodalton; L, liters; mAb, monoclonal antibody; mg, milligram; mg/kg, milligram per kilogram; MOA, mechanism of action; MW, molecular weight; n, sample size; nr-axSpA, non-radiographic axial spondyloarthritis; NSAID, non-steroidal anti-inflammatory drug; PK, pharmacokinetics; Q2W, every 2 weeks; Q4W, every 4 weeks; r-axSpA, radiographic axial spondyloarthritis; SC, subcutaneous; t_1/2_, half-life; Vd, volume of distribution.

Ixekizumab also demonstrated strong efficacy in r-axSpA. In COAST-V, ASAS40 response at week 16 was 52% with every 2-week dosing, 48% with every 4-week dosing, 36% with adalimumab, and 19% with placebo, with benefits maintained through week 52. Additionally, ixekizumab reduced spinal and sacroiliac MRI inflammation, with improvements in SPARCC scores sustained over time (Dougados et al., 2020). Beyond improvements in inflammatory MRI findings, a 52-week *post hoc* analysis of the COAST-V trial demonstrated that ixekizumab was associated with reduced sacroiliac joint erosion and increased backfill on SPARCC structural MRI scores, highlighting the emerging role of structural MRI endpoints as complementary measures to conventional inflammatory outcomes in r-axSpA (Maksymowych et al., 2025). In COAST-W, which enrolled TNF inhibitor experienced patients, ASAS40 response at week 16 was 31% with every 2-week dosing, 25% with every 4-week dosing, and 14% with placebo, with sustained responses through week 52 (Dougados et al., 2020; Marzo-Ortega et al., 2020b).

Bimekizumab has extended IL-17 pathway inhibition to both IL-17A and IL-17F. In BE MOBILE 1 and BE MOBILE 2, ASAS40 response at week 16 was 47.7% *versus* 21.4% with placebo in nr-axSpA and 44.8% *versus* 22.5% in r-axSpA, respectively (van der Heijde et al., 2023). These responses were sustained through 52 weeks, with additional improvement in enthesitis and peripheral arthritis among patients with musculoskeletal involvement (Baraliakos et al., 2025; Ramiro et al., 2025).

#### 5.2.3 Safety considerations

IL-17 plays a key role in mucocutaneous defense against *Candida* albicans and in maintaining intestinal epithelial barrier integrity (Conti and Gaffen, 2015). Pharmacologic inhibition therefore increases the class specific risk of mucocutaneous candidiasis and may exacerbate subclinical IBD (Bilal et al., 2024; Grümme et al., 2024). In the bimekizumab phase 2b BE AGILE study, 16 of 303 patients (5.3%) developed oral candidiasis over 48 weeks (van der Heijde et al., 2020). All cases were mild to moderate and did not lead to treatment discontinuation. Longer-term extension data from BE AGILE and subsequent integrated safety analyses further characterize the candidiasis safety profile of bimekizumab. *Candida* infections remained predominantly mild to moderate, localized, and non-invasive, with no reports of systemic fungal infection or emergence of new major safety signals during extended follow-up. In the 5-year BE AGILE open-label extension in r-axSpA, the exposure-adjusted incidence rate of *Candida* infection was 2.6 per 100 patient-years, while the corresponding rate for oral candidiasis was 2.2 per 100 patient-years, with no systemic fungal infections reported and treatment discontinuation due to candidiasis remaining uncommon (Deodhar et al., 2025). Consistent with these findings, an updated pooled safety analysis of six phase IIb/III studies in axSpA and psoriatic arthritis demonstrated that oral candidiasis remained predominantly mild to moderate, with an exposure-adjusted incidence rate of 3.5 per 100 patient-years in axSpA and very few cases leading to treatment discontinuation (0.2 per 100 patient-years), while serious opportunistic infections remained rare and no new safety signals were identified (Mease et al., 2026). From a clinical perspective, these findings indicate that dual IL-17A/IL-17F blockade confers a predictable and generally manageable risk of localized *Candida* infections that can usually be treated without permanent withdrawal of therapy. Importantly, clinical efficacy remained durable throughout long-term follow-up, with sustained improvements in ASAS40 responses, ASDAS low disease activity and inactive disease, BASDAI, and physical function, supporting a favorable long-term benefit-risk profile for bimekizumab (Deodhar et al., 2025). Comparable rates have been reported with secukinumab (1.7%) and ixekizumab (3.3%) across psoriasis and psoriatic arthritis trials (Saunte et al., 2017). Most cases resolve with standard antifungal therapy, including topical or systemic azoles, while continuing biologic treatment; discontinuation due to candidiasis is rare, affecting less than 1% of patients (Deodhar et al., 2025).

IL-17 inhibitors also carry a rare risk of new onset or worsening IBD (Deng et al., 2023). In a pooled safety analysis of 21 secukinumab trials including 7,355 patients (794 with AS), IBD events were uncommon, with exposure adjusted incidence rates in AS of approximately 0.2–0.8 per 100 patient years (Schreiber et al., 2019). Anti-TNF monoclonal antibodies remain the preferred choice over IL-17 inhibitors for patients with active or significant IBD history (Ramiro et al., 2023).

In contrast to TNF inhibitors, IL-17 inhibitors carry substantially lower tuberculosis reactivation risk. A pooled cohort study of 12,319 patients with psoriasis, psoriatic arthritis, or AS treated with secukinumab for up to 5 years reported no cases of active tuberculosis or latent tuberculosis infection reactivation (Elewski et al., 2021). Among AS patients, approximately 15.3% screened positive for latent tuberculosis infection and received chemoprophylaxis, with none developing active disease during follow up (Elewski et al., 2021). *In vitro* studies using a human three-dimensional microgranuloma model showed that secukinumab did not reactivate dormant *Mycobacterium tuberculosis*, unlike anti-TNF therapy (Kammüller et al., 2017).

### 5.3 Janus kinase inhibitors

#### 5.3.1 Mechanism of action

The JAK-STAT pathway mediates intracellular signaling for multiple cytokines implicated in AS pathogenesis, including IL-6, IL-12, IL-23, interferon-γ, and GM-CSF (Morris et al., 2018). Upon cytokine binding, receptor-associated JAKs (JAK1, JAK2, JAK3, TYK2) activate and phosphorylate STAT proteins (STAT1-6) (Xue et al., 2023). Activated STAT dimers translocate to the nucleus and drive transcription of inflammatory mediators and proliferation genes (Villarino et al., 2015).

JAK-STAT involvement in AS is supported by genetic evidence, with STAT3 polymorphisms linked to disease susceptibility and biologic response. Notably, IL-23 signals through JAK2/TYK2 to activate STAT3, while IL-17 receptor engagement recruits JAK-STAT signaling *via* Act1 adaptor protein (Pastor-Fernández et al., 2020). By targeting these intracellular cascades, JAK inhibitors provide complementary inhibition to biologics that block extracellular cytokines (Figure 4C).

#### 5.3.2 Clinical efficacy

The pivotal SELECT-AXIS 1 trial demonstrated that upadacitinib significantly improved clinical outcomes in biologic-naïve patients with active AS (van der Heijde et al., 2019). In this trial, ASAS40 response at week 14 was 52%–54% with upadacitinib 15 mg *versus* 26% with placebo. Long-term data through 104 weeks showed sustained and improving responses, with ASAS40 rates reaching 72% (non-responder imputation) and 85% (as observed) at week 64 (Deodhar et al., 2022a). Similar efficacy was observed in SELECT-AXIS 2 (420 patients refractory to 1–2 biologics), upadacitinib achieved ASAS40 response of 45% *versus* 18% placebo at week 14 (*p*<0.0001) (van der Heijde et al., 2022). Upadacitinib also significantly improved secondary endpoints including ASAS20, ASDAS clinically important improvement, BASDAI50, back pain, enthesitis (MASES), function (BASMI), quality of life (ASQoL), and MRI inflammation scores (SPARCC spine/sacroiliac joint) (van der Heijde et al., 2022). Table 3 summarizes the clinical and pharmacological profiles of these oral agents, comparing their selectivity, dosing regimens, and current regulatory approval status.

**TABLE 3:** JAK inhibitors in ankylosing spondylitis.

Drug	Structure	MW (Da)	MOA	Dose	PK	Key outcomes	Notes	FDA approval	References
Tofacitinib	First generation; low molecular weight ATP-competitive small molecules	312.37	Inhibits JAK1/JAK3 > JAK2; blocks ATP binding to catalytic site	5 mg orally BID (IR), or 11 mg OD (ER)	t_1/2_ 3 h; BA 74%; peak at 0.5–1 h; steady state within 24–48 h	Phase 2: ASAS20 80.8% vs. 41.2% placebo; ASAS40 48.4% vs. 20.0%	Inhibits TYK2 at higher concentrations	2021	(van der Heijde et al., 2017; Deodhar et al., 2023)
Upadacitinib	Second generation; low molecular weight ATP-competitive small molecule	380.38	Selective JAK1 inhibition (>40-fold) vs. JAK3	15 mg orally OD (ER)	t_1/2_ 8–14 h; BA 70%–80%; peak 2–4 h; steady state 4 days	SELECT-AXIS 1: ASAS40 51.6% vs. 25.5% placebo (biologic-naïve), reaching 72% (non-responder imputation) and 85% (as observed) at week 64 SELECT-AXIS 2: ASAS40 at week 14 achieved 45% vs. 18% placebo (biologic-refractory) At week 14: ASAS20 65% vs. 38% placebo, ASDAS clinically important improvement 62% vs. 22%, ASDAS inactive disease 13% vs. 2%, SPARCC MRI spine inflammation −3.95 vs. −0.04, SPARCC MRI sacroiliac joint −2.26 vs. 1.05, BASDAI50 43% vs. 17%, total back pain −3.0 vs. −1.47, MASES -2.6 vs. −1.1, BASMI -0.48 vs. −0.16, ASQoL −5.10 vs. −2.03	High JAK1 selectivity	2022	(Deodhar et al., 2022a; van der Heijde et al., 2022; Baraliakos et al., 2023)
Filgotinib	Second generation; low molecular weight ATP-competitive small molecule	425.51	Selective JAK1 inhibition	200 mg oral OD	t_1/2_ 7 h (19 h active metabolite); peak 2–3 h; steady state 2–4 days	Phase 2 TORTUGA at week 12 showed significant improvements: ASDAS mean change was −1.47 vs. −0.57 placebo, ASAS20 achieved 76% vs. 40% placebo, ASAS40 38% vs. 19% placebo, ASAS partial remission in 12% vs. 3% placebo, BASMI significantly improved −0.75 vs. −0.39, SPARCC spine inflammation scores decreased by 5.76 points vs. an increase of 0.52 placebo, and SPARCC sacroiliac joint of −3.52 vs. 0.06	High JAK1 inhibition	Not approved by the FDA for any indication, as phase 3 clinical trials in the United States were discontinued due to concerns related to male reproductive safety	(van der Heijde et al., 2018b; Honap et al., 2023)
Ivarmacitinib	Second-generation; low molecular weight ATP-competitive small molecule	414.49	Selective JAK1 inhibition	4 mg orally OD	Not reported in published literature	Phase 2/3: ASAS20 48.7% vs. 29.0%; ASAS40 32.1% vs. 18.3% at week 12	High JAK1 selectivity	Not FDA approved; regulatory approval is currently limited to China	(Liu et al., 2025; Keam, 2025)

This table summarizes oral JAK inhibitors, offering convenient administration compared to injectables. Tofacitinib and upadacitinib achieve ASAS40 responses of 40%–52% across biologic-naïve and experienced patients, while filgotinib showed significant improvements in phase 2. Cardiovascular, thromboembolic, and malignancy risk monitoring is essential.

AS, ankylosing spondylitis; ASAS, Assessment of Spondyloarthritis International Society; ASAS20, Assessment of Spondyloarthritis International Society 20%; ASAS40, Assessment of Spondyloarthritis International Society 40%; ASDAS, Ankylosing Spondyloarthritis Disease Activity Score; ASQoL, Ankylosing Spondylitis Quality of Life; ATP, adenosine triphosphate; BA, bioavailability; BASDAI50, Bath Ankylosing Spondylitis Disease Activity Index 50% response; BASMI, Bath Ankylosing Spondylitis Metrology Index; BID, twice daily; Da, dalton; ER, extended-release; FDA, Food and Drug Administration; hr, hour; IR, immediate-release; JAK, Janus kinase; JAK1/2/3, Janus kinase 1, 2, or 3; MASES, Maastricht Ankylosing Spondylitis Enthesitis Score; MOA, mechanism of action; MRI, magnetic resonance imaging; MW, molecular weight; NSAID, non-steroidal anti-inflammatory drug; OD, once daily; PK, pharmacokinetics; SPARCC, Spondyloarthritis Research Consortium of Canada; STAT, signal transducer and activator of transcription; t1/2, half-life; TYK2, tyrosine kinase 2.

Tofacitinib showed promising results in phase 2, with ASAS20 response of 80.8% *versus* 41.2% placebo and ASAS40 response of 48.4% *versus* 20.0% in active AS (van der Heijde et al., 2017). Phase 3 data confirmed efficacy in broader populations.

Filgotinib demonstrated significant improvements in the phase 2 TORTUGA trial, including ASDAS response, BASMI, and MRI inflammation (SPARCC spine/sacroiliac scores) in active AS (van der Heijde et al., 2018b).

More recently, ivarmacitinib, a highly selective JAK1 inhibitor, has demonstrated promising efficacy in patients with active AS. In a phase 2/3 trial involving patients with an inadequate response to NSAIDs, once-daily ivarmacitinib 4 mg achieved ASAS20 and ASAS40 response rates of 48.7% and 32.1% at week 12, compared with 29.0% and 18.3% placebo. Clinical improvements were sustained through week 24, and the treatment was generally well tolerated with a manageable safety profile. These findings further expand the therapeutic landscape of selective JAK1 inhibition in axSpA (Liu et al., 2025).

#### 5.3.3 Safety considerations

JAK inhibitors require routine monitoring of complete blood count, liver function, renal function, and lipid profile. Upadacitinib 15 mg once daily is associated with dose dependent creatine phosphokinase elevations, which occur more frequently than with methotrexate or adalimumab but are typically asymptomatic, transient, and rarely lead to discontinuation (Charles-Schoeman et al., 2024).

Safety concerns emerged from rheumatoid arthritis trials, particularly the ORAL Surveillance study of 4,362 patients aged 50 years or older with at least one cardiovascular risk factor randomized to tofacitinib *versus* TNF inhibitors (Ytterberg et al., 2022). After 4 years of median follow up, tofacitinib showed numerically higher rates of major adverse cardiovascular events (hazard ratio 1.33, 95% CI 0.91-1.94), venous thromboembolism, and all-cause mortality compared with TNF inhibitors, although absolute event rates remained low. These findings led to FDA boxed warnings for all JAK inhibitors regarding serious cardiovascular events, thrombosis, malignancy, and mortality risks (U.S. Food and Drug Adminstration F, 2021). While caution is warranted, AS patients are typically younger with different cardiovascular risk profiles than the ORAL Surveillance population. Nevertheless, careful patient selection and risk assessment remain essential, particularly for patients aged 50 years or older or those with obesity, prior venous thromboembolism, or other thrombotic risk factors.

Furthermore, long-term safety has also been evaluated in the integrated SELECT clinical trial program, which included 1,789 patients with psoriatic arthritis, AS, and nr-axSpA, representing 3,689 patient-years of upadacitinib exposure over up to 5 years of follow-up. Overall, the safety profile remained consistent with previous reports, with no new safety signals identified. Rates of adjudicated major adverse cardiovascular events, venous thromboembolism, and malignancy excluding non-melanoma skin cancer were low and comparable across disease indications, whereas higher rates of serious infection, herpes zoster, lymphopenia, and non-melanoma skin cancer were observed in patients with psoriatic arthritis than in those with AS or nr-axSpA (Burmester et al., 2024). These findings provide reassurance that the long-term safety profile of upadacitinib in axSpA has remained stable in clinical trials while reinforcing the importance of individualized patient selection and ongoing safety monitoring.

An additional class-specific safety consideration is the increased risk of herpes zoster associated with JAK inhibition, with approximately double the risk compared with placebo or TNF inhibitors. In SELECT-AXIS 2, herpes zoster incidence with upadacitinib was 3.8 events per 100 patient years through 104 weeks, with one serious case (0.1 per 100 patient years) (Baraliakos et al., 2024b). This risk is particularly high in older patients and those receiving concomitant glucocorticoids or other immunosuppressive therapies. Accordingly, current rheumatology guidelines recommend administration of the recombinant zoster vaccine (RZV) before or during treatment with JAK inhibitors. As a non-live vaccine, RZV can be safely administered to immunosuppressed adults and represents an important strategy for reducing herpes zoster risk (Anderson et al., 2022; Bass et al., 2022). Therefore, regulatory guidance emphasizes both cardiovascular risk assessment and herpes zoster vaccination before treatment initiation (EMA, 2022).

### 5.4 Therapeutic considerations for non-radiographic axial spondyloarthritis

Within the broader axSpA spectrum, several biologic and targeted synthetic DMARDs have demonstrated robust efficacy in nr-axSpA, although regulatory eligibility criteria and sequencing strategies may differ from radiographic disease. In the pivotal ABILITY-1 trial of adalimumab 40 mg every other week in adults fulfilling ASAS axial SpA criteria but not the modified New York criteria, 36% of patients with active nr-axSpA achieved ASAS40 at week 12 compared to 15% in the placebo group (Sieper et al., 2013), with long-term open-label follow-up showing sustained clinical remission (ASDAS inactive disease at all visits) in 24% of patients during year 2 (weeks 52–104), 23% during year 3 (weeks 104–156), and 18% across both years (van der Heijde et al., 2018c). Earlier randomized data in axSpA without radiographically defined sacroiliitis similarly demonstrated that 54.5% of patients receiving adalimumab achieved ASAS40 at week 12 *versus* 12.5% on placebo (Haibel et al., 2008), underscoring the effectiveness of TNF-α blockade in nr-axSpA. Other TNF inhibitors, including etanercept and certolizumab pegol, have shown comparable improvements in ASAS20, ASAS40 and BASDAI50 in nr-axSpA, with one meta-analysis reporting that adalimumab, etanercept, and certolizumab pegol were all effective and well tolerated, and that patients were more likely to reach ASAS20 and ASAS40 with etanercept or adalimumab than with placebo, with the lowest number needed to treat observed for adalimumab (Olivieri et al., 2016).

More recently, IL-17A inhibition has been validated in nr-axSpA: in the phase III PREVENT trial, secukinumab 150 mg achieved ASAS40 in 41.5% of TNF-inhibitor naïve nr-axSpA patients at week 16 *versus* 29.2% with placebo, with particularly high response rates of approximately 52.3% *versus* 21.8% in patients who had both elevated CRP and MRI-positive sacroiliitis (Braun et al., 2021). In the COAST-X trial, ixekizumab 80 mg every 4 weeks or every 2 weeks yielded ASAS40 response rates of 35%–40% at week 16 compared with 19% on placebo and sustained responses of 30%–31% *versus* 13% at week 52, leading to regulatory approval for active nr-axSpA with objective signs of inflammation (Deodhar et al., 2020). Emerging data also support other IL-17 inhibitors; in a Japanese phase III study including patients with AS and nr-axSpA, brodalumab achieved week 16 ASAS40 in 43.8% of patients *versus* 24.1% on placebo, suggesting it may represent a future therapeutic option in axSpA (Kirin, 2020).

Beyond biologic agents, the JAK-inhibitor upadacitinib has become the first targeted synthetic DMARD approved specifically for active nr-axSpA with objective signs of inflammation: in the SELECT-AXIS 2 trial, upadacitinib produced significantly higher ASAS40 responses than placebo and clinically meaningful improvements in pain, function, and MRI/CRP inflammatory activity in patients with inadequate response or intolerance to TNF inhibitors (Deodhar et al., 2022b). Overall, systematic literature reviews and network meta-analyses indicate that TNF monoclonal antibodies and IL-17 inhibitors provide similar magnitudes of benefit in nr-axSpA and r-axSpA, with ASAS40 numbers needed to treat generally in the range of approximately 2–5 compared with placebo when objective signs of inflammation are present (Sepriano et al., 2017).

## 6 Treatment sequencing strategies

Biologic initiation is recommended for patients with persistently high axSpA disease activity despite adequate trials of ≥2 different NSAIDs at maximum tolerated anti-inflammatory doses for ≥2–4 weeks, per 2022 ASAS-EULAR recommendations. High disease activity is defined as ASDAS ≥2.1 (or BASDAI ≥4 with objective inflammation: elevated CRP, MRI sacroiliac inflammation, or radiographic sacroiliitis meeting modified New York criteria) (Ramiro et al., 2023).

First-line biologic selection supports either TNF inhibitors or IL-17 inhibitors, with JAK inhibitors as an alternative based on patient characteristics and comorbidities (Ramiro et al., 2023). Therapy choice considers overlapping efficacy across axial, peripheral, and extra-articular manifestations, with certain agents demonstrating superior effectiveness for specific features.

Therapeutic choice should be guided by the dominant clinical phenotype. For instance, recurrent acute anterior uveitis or active IBD generally favors TNF monoclonal antibodies such as adalimumab, infliximab, golimumab, certolizumab pegol because of their dual axial disease and extra-articular efficacy (Ramiro et al., 2023; Fabiani et al., 2016). Conversely, IL-17 inhibitors (secukinumab, ixekizumab, bimekizumab, brodalumab where available) are preferred in patients with significant psoriasis (Torres et al., 2024), while prominent peripheral enthesitis or arthritis supports the use of bimekizumab due to its high-resolution rates (>40% complete enthesitis resolution and >60% peripheral arthritis resolution at 104 weeks in BE MOBILE 1/2) (Ramiro et al., 2025).

Moreover, inadequate response is defined as failure to achieve ASDAS improvement ≥1.1 (clinically important improvement) after ∼12 weeks of optimized biologic dosing constitutes primary failure. Switching after treatment failure should be individualized to each patient’s factors and disease progression. Primary failure to one TNF inhibitor would encourage switching to a second TNF inhibitor in some patients (class cycling), although registry data suggest that response rates may be lower than in biologic-naïve populations. If clinically relevant improvement is still absent, switching to an IL-17 inhibitor is recommended (Ramiro et al., 2023). JAK inhibitors are also viable post-biologic DMARD failure; SELECT-AXIS 2 demonstrated upadacitinib ASAS40 response of 45% vs. 18% placebo at week 14 in biologic-refractory patients (van der Heijde et al., 2022).

Accordingly, early treatment optimization should be prioritized over prolonged trial-and-error switching, as persistent inflammation may contribute to cumulative disease burden and reduce the likelihood of achieving durable control. Timely escalation to an effective agent may therefore help preserve function for as long as possible and improve the overall quality of long-term disease management. In addition, patient-centered shared decision-making should be integrated into treatment selection since route of administration, monitoring requirements, and patient preference can substantially influence adherence and real-world effectiveness.

Health economics evidence suggests that biologic strategy selection should be interpreted with caution, as cost-effectiveness is highly sensitive to assumptions about response rates, treatment duration, drug pricing, and the threshold used for willingness to pay. A patient-level simulation modeling over 10 years found sequential TNF inhibitor followed by IL-17A inhibitor therapy was considered cost-effective at $150,000/QALY threshold ($179,118 total discounted cost, 5.893 QALYs/patient). Whereas no biologic strategy reached cost-effectiveness at $100,000/QALY *versus* conventional therapy (Le et al., 2020). This implies that even highly effective biologics may remain economically challenging in stricter reimbursement environments, especially when long-term treatment is required. This is an important reminder that clinical efficacy alone does not fully determine therapeutic value. In AS, the most defensible economic strategy is likely one that combines evidence-based sequencing, early identification of the right biologic for the right phenotype, and broader access to biosimilars where appropriate. That approach is more likely to improve both patient outcomes and system sustainability than a strategy based only on the lowest upfront acquisition cost.

## 7 Biosimilars in ankylosing spondylitis

**FIGURE 5:**
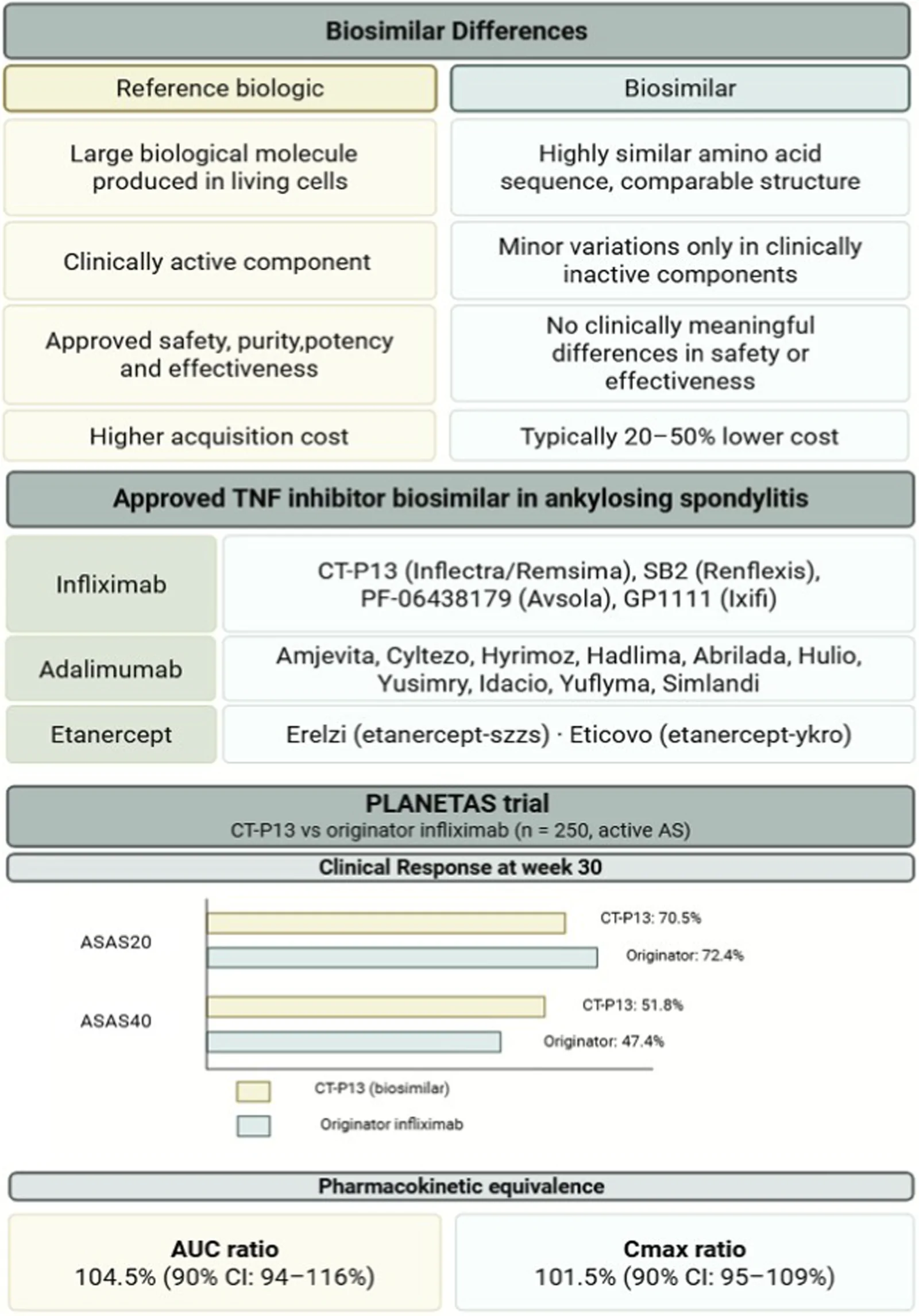
Biosimilars in ankylosing spondylitis. First panel provides a schematic comparison between reference biologics and biosimilars across four domains: molecular structure, composition, clinical outcomes, and cost. Biosimilars are highly similar to their reference products in amino acid sequence and structure, with minor variations confined to clinically inactive components and no clinically meaningful differences in safety or effectiveness, typically offering 20%–50% lower acquisition costs. Second panel lists all approved TNF inhibitor biosimilars for AS, grouped by originator: four infliximab biosimilars (CT-P13, SB2, PF-06438179, GP1111), ten adalimumab biosimilars, and two etanercept biosimilars, all maintaining identical dosing regimens and routes of administration to their originator compounds. Third panel presents key efficacy and pharmacokinetic data from the pivotal PLANETAS trial (Phase 1, randomized, double-blind, multicenter; n = 250), demonstrating comparable ASAS20 (CT-P13 70.5% vs. originator 72.4%) and ASAS40 (CT-P13 51.8% vs. originator 47.4%) response rates at week 30, with pharmacokinetic equivalence confirmed by an AUC ratio of 104.5% (90% CI: 94%–116%) and Cmax ratio of 101.5% (90% CI: 95%–109%). Figure is adapted and modified from Park et al. (2013) and Yoo et al. (2013), created using software tools of BioRender.com. AS, ankylosing spondylitis; ASAS20/40, Assessment of SpondyloArthritis International Society 20%/40% improvement criteria; AUC, area under the concentration-time curve; CI, confidence interval; Cmax, maximum serum concentration; TNF, tumor necrosis factor.

Biosimilars of TNF inhibitors provide clinically equivalent alternatives to originator biologics for the treatment of AS, demonstrating comparable efficacy, safety, pharmacokinetic profiles, and dosing regimens. By offering potentially lower-cost treatment options, they may also enhance patient access to biologic therapy. The key approved agents and the clinical evidence supporting their use are summarized in Figure 5.

## 8 Clinical monitoring and patient management

In addition to control of disease activity, symptoms that require recognition and appropriate treatment include fatigue, sleep disturbance, psychological distress, and pain-related limitations that contribute to reduced quality of life. Active patient engagement in shared decision-making with their clinical team regarding treatment goals, risks, and preferences is central to personalized management in axSpA. Patients vary greatly in their disease pattern, response to different therapies, rate of disease progression, and goals and preferences (Ramiro et al., 2023).

Disease activity should be regularly assessed using standardized validated measures. The ASDAS with C-reactive protein (ASDAS-CRP) is recommended as the preferred instrument for monitoring disease activity in axSpA due to its improved validity and incorporation of an objective inflammatory marker, whereas BASDAI may be used as an alternative. ASDAS is calculated using patient-reported measures of back pain (0–10 scale), duration of morning stiffness (0–10 scale), patient global assessment (0–10 scale), peripheral pain/swelling (0–10 scale), and CRP (mg/L). ASDAS-CRP inactive disease is defined as score <1.3, low disease activity as ≥1.3 to <2.1, high disease activity as ≥2.1 to ≤3.5, and very high disease activity as >3.5. An ASDAS change of ≥1.1 represents clinically important improvement, and ≥2.0 represents major improvement (Wiąk-Walerowicz and Wielosz, 2024).

Response to biologic therapy should be assessed at baseline and at least 12 weeks after initiation. Treatment continuation should be based on evidence of clinically important improvement, preferably an ASDAS decrease ≥1.1, together with clinical judgement regarding overall response and patient wellbeing. Ongoing monitoring should be conducted regularly to assess sustained response, detect disease flares, and safety, with visit frequency depending on individual patient case according to disease activity, severity, and treatment (Ramiro et al., 2023). Additionally, based on international expert consensus treat-to-target recommendations, monitoring is typically performed within 3 months, more frequently if treatment targets are not achieved, and every 6–12 months once disease control is established (Beckers et al., 2022).

Before initiating biologic therapy, comprehensive baseline assessment should include tuberculosis screening using tuberculin skin test (≥5 mm induration considered positive in high-risk groups such as immunocompromised patients) or interferon-gamma release assays (QuantiFERON-TB Gold or T-SPOT.TB), chest radiography to detect prior tuberculosis or active pulmonary disease, and detailed tuberculosis exposure history. Hepatitis B screening includes hepatitis B surface antigen, hepatitis B core antibody (total), and hepatitis B surface antibody; hepatitis C screening includes hepatitis C antibody with reflex HCV RNA testing if positive. Pregnancy testing *via* serum or urine beta-hCG in women of childbearing potential is required prior to initiation of biologic therapy (Fragoulis et al., 2022; Holroyd et al., 2019). Baseline imaging includes plain radiographs of the sacroiliac joints and spine to assess structural damage, with repeat radiographs at least every 2 years in patients with risk factors for progression (baseline syndesmophytes, male gender, smoking, elevated acute-phase reactants, physically demanding occupation) (Maas et al., 2017).

During biologic therapy, disease activity assessment should be evaluated at each visit using validated measures, at 3–4 months after initiation, and subsequently every 6–12 months (Zhao SS. et al., 2025). Laboratory monitoring includes CRP if elevated at baseline or clinically indicated to assess inflammatory burden and treatment response, CBC every 3–6 months (more frequently for JAK inhibitors), hepatic transaminases and creatinine every 3–6 months, and lipid profile at baseline, 12 weeks after initiation, and every 6–12 months for JAK inhibitors (Emer et al., 2010; Kirchhof et al., 2024). Safety monitoring requires systematic inquiry regarding symptoms of infection (fever, cough, dysuria, skin infections), with particular attention to respiratory symptoms and tuberculosis exposure for TNF inhibitor-treated patients. For IL-17 inhibitor-treated patients, monitoring for signs of mucocutaneous candidiasis (oral thrush, esophageal candidiasis, vulvovaginal candidiasis) and gastrointestinal symptoms potentially suggestive of IBD (chronic diarrhea, abdominal pain, rectal bleeding) is recommended.

## 9 Limitations and future considerations

Although treatment options for AS have expanded considerably, several limitations remain. Most evidence comes from controlled trials with selected patient groups, so results may not fully reflect everyday clinical practice. In addition, indirect comparisons across drug classes are difficult because studies differ in design, patient populations, and outcome measures.

### 9.1 Evolving safety profiles

Long-term safety is also still evolving, especially for newer IL-17 and JAK-targeted therapies. TNF inhibitors have the most established safety record, but ongoing monitoring is still important for infections and other rare adverse effects.

Another limitation is the lack of reliable biomarkers that can predict which patient will respond best to a specific treatment. At present, drug choice still depends mainly on clinical features such as psoriasis, uveitis, IBD, prior response, and safety concerns. This makes treatment selection more empirical than truly personalized.

Future research should focus more on direct comparison between drug classes, longer real-world follow-up, and better tools for predicting treatment response. More studies are also needed on radiographic progression, sequencing strategies after treatment failure, and the long-term impact of early biologic use. Cost-effectiveness and access to newer therapies and biosimilars will also remain important as treatment options continue to grow. Table 4 highlights suggested research areas that need to be visited by researchers worldwide. The table highlights key evidence gaps in AS management, including the need for head-to-head comparisons, real-world data, and long-term safety and progression outcomes. It also underscores the importance of optimizing treatment sequencing, advancing biomarker-driven precision medicine, and addressing cost-effectiveness to improve access.

**TABLE 4:** Research priorities in the management of ankylosing spondylitis: suggested gaps and future directions.

Category	Priority research need
Comparative effectiveness	Head-to-head trials comparing TNF, IL-17, and JAK inhibitors
Real-world evidence	Real-world effectiveness studies beyond controlled trial populations
Disease progression	Radiographic progression data for newer agents
Treatment strategy	Optimal sequencing strategies post-primary/secondary failure
Precision medicine	Biomarker discovery for treatment response prediction
Safety	Long-term safety in AS-specific populations
Health economics	Cost-effectiveness analyses incorporating biosimilars and access barriers

## 10 Conclusion

The management of AS has undergone a fundamental shift toward targeted, mechanism-based therapy driven by advances in the understanding of disease immunopathology. Biologic and targeted synthetic DMARDs directed against TNF-α, the IL-17 pathway, and JAK-STAT signaling now form the cornerstone of treatment for patients with active disease refractory to NSAIDs, offering consistent reductions in inflammation, improvement in function, and sustained long-term disease control.

Among these therapies, TNF inhibitors remain a well-established first-line biologic option with extensive long-term data and proven efficacy for extra-articular manifestations such as uveitis and IBD. Similarly, IL-17 inhibitors provide comparable efficacy for axial disease and are particularly advantageous in patients with significant psoriasis or increased tuberculosis risk, while dual IL-17A/F inhibition may offer enhanced control of enthesitis and peripheral arthritis. JAK inhibitors add an effective oral therapeutic option across both biologic-naïve and biologic-experienced populations, although careful cardiovascular and thrombotic risk assessment is essential.

Optimal outcomes in AS depend on a personalized, targeted approach using validated disease activity measures, objective evidence of inflammation, and thoughtful consideration of comorbidities and patient preferences. Rational sequencing across therapeutic classes and the expanding availability of biosimilars have further improved treatment accessibility and cost-effectiveness. Continued research into structural progression, predictive biomarkers, and novel targets will be critical to further refining long-term management strategies.
